# My Corporis Fabrica Embryo: An ontology-based 3D spatio-temporal modeling of human embryo development

**DOI:** 10.1186/s13326-015-0034-0

**Published:** 2015-09-24

**Authors:** Pierre-Yves Rabattu, Benoit Massé, Federico Ulliana, Marie-Christine Rousset, Damien Rohmer, Jean-Claude Léon, Olivier Palombi

**Affiliations:** Department of Anatomy, LADAF, Université Joseph Fourier, Grenoble, France; LJK (CNRS-UJF-INPG-UPMF), INRIA, Université de Grenoble, Grenoble, France; LIG (CNRS-UJF-INPG-UPMF), Université de Grenoble, Grenoble, France; CPE Lyon, Université de Lyon, Lyon, France

## Abstract

**Background:**

Embryology is a complex morphologic discipline involving a set of entangled mechanisms, sometime difficult to understand and to visualize. Recent computer based techniques ranging from geometrical to physically based modeling are used to assist the visualization and the simulation of virtual humans for numerous domains such as surgical simulation and learning. On the other side, the ontology-based approach applied to knowledge representation is more and more successfully adopted in the life-science domains to formalize biological entities and phenomena, thanks to a declarative approach for expressing and reasoning over symbolic information. 3D models and ontologies are two complementary ways to describe biological entities that remain largely separated. Indeed, while many ontologies providing a unified formalization of anatomy and embryology exist, they remain only descriptive and make the access to anatomical content of complex 3D embryology models and simulations difficult.

**Results:**

In this work, we present a novel ontology describing the development of the human embryology deforming 3D models. Beyond describing how organs and structures are composed, our ontology integrates a procedural description of their 3D representations, temporal deformation and relations with respect to their developments. We also created inferences rules to express complex connections between entities. It results in a unified description of both the knowledge of the organs deformation and their 3D representations enabling to visualize dynamically the embryo deformation during the Carnegie stages. Through a simplified ontology, containing representative entities which are linked to spatial position and temporal process information, we illustrate the added-value of such a declarative approach for interactive simulation and visualization of 3D embryos.

**Conclusions:**

Combining ontologies and 3D models enables a declarative description of different embryological models that capture the complexity of human developmental anatomy. Visualizing embryos with 3D geometric models and their animated deformations perhaps paves the way towards some kind of hypothesis-driven application. These can also be used to assist the learning process of this complex knowledge.

**Availability:**

http://www.mycorporisfabrica.org/

**Electronic supplementary material:**

The online version of this article (doi:10.1186/s13326-015-0034-0) contains supplementary material, which is available to authorized users.

## Background

Embryology has been studied for many centuries. This morphologic discipline studies the transformation of a single cell into a complete organism, which is composed of almost 10^14^ cells. This discipline has benefited of progress from imagery, histology, molecular biology and genetic. However it remains a high level of complexity and lots of physiological and pathological mechanisms stay unclear.

Computer modeling and simulation of human body allow to express and to visualize complex physiological processes, to make the steps of human embryology more accessible. However, no unified model which allows integrating embryological processes into 3D visualization exists.

An ontology is a formal description of a domain of interest [1]. Ontologies are increasingly employed in the life-science domains, and in particular in biology, for the sake of standardizing a common vocabulary of entities and expressing the inherent complexity of biological systems. Moreover, ontologies formal systems can be read, understood and exploited by computers to carry out tasks that would be otherwise tedious and time-consuming for the humans. The main biology ontologies are referenced in OBO Foundry [2] and NCBO Bioportal [3].

Human anatomy ontologies are referenced by the Foundational Model of Anatomy domain, FMA [4], which contains the concepts and relationships that pertain to the structural organization of the human body. Uberon [5] is a multi-species ontology for anatomy (just as the gene ontology (GO) project [6] is a multi-species ontology for molecular functions, biological processes or cellular components). It is integrated with species-specific ontologies, but it does not dependent on them.

Regarding embryology, some interesting concepts have been introduced in several anatomical ontologies like the drosophila (DAO) one [7]. More specifically, Kaufman [8] developed the Atlas of Mouse Development which has been integrated into the Mouse Atlas Project (MAP) led by Davidson [9], Bard [10], Baldock [11] and Ringwald [12]. It is a study of mouse developmental anatomy through histological sections of mouse embryos reconstructed in a full-grey image, for each developmental stage (Theiler stages). Brune [13] has completed this project by defining 3D regions (domains) of the embryo models, to map an ontology to each of these spatial models. Through the 3D domains, users can navigate from the spatial representation of the embryo to the ontology and vice versa. It is important to note that there is one ontology for each Theiler stage. Links between ontologies and 3D images also exist in some other atlas as in the Virtual Fly Brain project allowing users to explore the structure of the *Drosophila* brain by browsing 3D images of a brain with subregions displayed as colored overlays [14] and, in a more general setting, in Allen Brain Atlas [15].

Burger [16] developed the concept of the “Abstract Mouse” which was further developed by Hayamizu [17] who created a non-stage specific representation of a mouse developmental anatomy ontology (EMAPA, [18]) where each anatomical entity is linked to the first and last stages of its existence.

Hunter [19] has built a human developmental anatomy ontology (EHDA), which is composed of several ontologies, one for each Carnegie stage (CS) [1–20] that only includes basic *part_of* data. Bard [20] developed an ontology of human developmental anatomy (EHDAA2) with more than 2000 anatomical entities, which are linked by *part_of, is_a, develops_from* relationships and linked to the Carnegie stage by *starts_at* and *ends_at* relations. He had initially developed a work, as part of the Edinburgh Mouse Atlas Project, to provide a structured and controlled vocabulary of stage-specific anatomical structures for the developing laboratory mouse.

Palombi et al. [21,22] have developed an ontology of human anatomy which includes 3D representations, where each anatomical entity is linked to its corresponding 3D model. They also considered the function of each entity to assist the creation of complex 3D models for visualization and simulation [23]. My Corporis Fabrica (MyCF) is equipped with automatic reasoning capabilities that enable model checking and complex queries answering. They demonstrated that 3D graphical models are effective to represent either anatomy or embryology.

This kind of 3D modeling is already used in Biomechanics, Computer Aided Medicine and Computer Graphics. It can be useful in plenty of domains like biomechanics, ergonomics, diagnosis, treatment planning, visualization, graphics, robotics, or, for example, helping deduce the anatomic consequences of an injury [24].

Kerwin et al. have developed a three-dimensional atlas of the human embryonic brain using anatomical landmarks and gene expression data to define major subdivisions through 12 stages of development (Carnegie Stages 12–23). Virtual 3D anatomical models were generated from intact specimens using optical projection tomography. The 3D models and a preliminary set of anatomical domains and ontology are available on the atlas pages along with gene expression data from approximately 100 genes in the HUDSEN Human Spatial Gene Expression Database [25,26].

Gasser [27] developed the Virtual Human Embryo [28], which is an atlas of digitally captured images of serial embryo sections corresponding to each of the 23 Carnegie stages. Then, they were assembled to provide 3D images and holistic views of development. Moreover, this database is accessible to all researchers, teachers and students and can be used acknowledging the source of the images, i.e., Virtual Human Embryo DREM Project.

Full exploitation of ontologies goes far beyond providing standardized notations for explicit formalized knowledge on domains of interest. It requires equipping ontological statements with reasoning and querying capabilities in order to efficiently retrieve relevant and precise information. However, until now, only few existing works in biomedical ontologies (e.g., [21–24]) take advantage of available automatic reasoners. Most of these works rely on ontologies expressed in OWL and use OWL reasoners such as Pellet [29] or Racer [30] that come with a high computational complexity in the worst case (as they are Description Logics reasoners). Recently, RDF-based semantic environments such as Jena (http://jena.apache.org/) or Cwm (http://www.w3.org/2000/10/swap/doc/cwm) have included rules (a la Datalog) to perform inferences on top of RDF datasets. Datalog rules [31] and Description Logics [32] are two orthogonal decidable fragments of first-order logics that have been extensively studied in knowledge representation and in deductive databases. The interest of Datalog rules is that they are easy to read and write for practitioners and they have a polynomial data complexity while allowing expressing complex interaction between properties and recursivity. In addition, as it will be shown in our examples, rules allows to capture in a uniform manner OWL constraints that are useful in practice, such as property transitivity or symmetry, but also domain-specific rules with practical relevance for users in many domains of interest.

In this paper, we describe a novel ontology of human embryo developmental anatomy. Thanks to a RDF-based uniform knowledge representation formalism, it enables interoperability between anatomical models (based on a stage-independent hierarchical structure like Bard’s), 3D graphical models [21] and spatio-temporal representations of development processes. This integrated declarative approach facilitates the generation of animations of the embryological development and opens new possibilities for learning and reasoning, in particular for a better understanding of developmental abnormalities. The key issue to achieve an effective interoperability is the rule-based inference allowing us to express quite simply how properties interact to relate the different models.

Our final goal is to include some malformation pathologies and represent them so as to increase the understanding of the mechanism of their pathogenesis. The embryological entities we consider have been willingly restricted in numbers and simplified in terminology to ease the development and the test of our approach.

## Results

The MyCF Embryo ontology has been designed so as to accommodate the description of several embryos, male or female, their 3D representations and their spatio-temporal developmental processes.

MyCF Embryo ontology is made of 6 taxonomies of classes related by relations, and by a set of 15 rules, that we will describe now.

The top classes of the different taxonomies are: embryological_entity, temporal_entity, geometrical_entity, process, spatio-temporal_representation and disease.

Each embryological entity is assigned to a set of spatio-temporal representations describing a geometrical component at a given time or during a process over a given duration.

We now describe the main classes in the ontology and the properties linking them. Additional file 1: Table S1 summarizes them and provides their definitions and the correspondences with existing ontologies if any.

Our ontology is expressed in RDFS [33] which is broadly used in Linked Data [34] to express so-called light-weight ontologies with a rule-based semantics. Whereas OWL is often seen as an extension of RDFS, this is not exactly the case, mainly because RDFS allows to use the same identifier as a class, instance or property, which is not possible in Description Logics (at the basis of OWL profiles), in which the sets of instances, classes and properties must be disjoint. In addition, RDF allows to use blank nodes in class or property position.

Similarly, the RDF query language SPARQL can query simultaneously the data and the schema and allows variables to stand for classes and properties. This goes beyond the first-order conjonctive queries typically considered in Description Logics. It is worth emphasizing that OWL and RDFS can interoperate, as there exists an RDF triple notation for most of the OWL constructs (in particular the OWL2 profiles), and the corresponding axioms can be expressed as logical rules. An important need was to express fine-grained domain-specific rules, in particular for describing the spatio-temporal development relations between subparts of the embryo. As it will be shown below, such rules are quite easy to express on top of RDF facts, while they are not expressible in Description Logics and thus, in OWL.

### Embryological_entity

Embryological entities model the anatomical structure of a formal embryo as classes (e.g., *kidney, left_kidney, embryo*) directly related by properties (that hold between classes and not between instances in contrast with modeling using OWL). Thus, declaring that left kidney is a part of embryo will be simply expressed by a single RDF triple *<left_kidney, part_of, embryo>*, as it is done in MyCF ontology [21].

Embryological entities can be material or immaterial. For example, the kidney, gonad and cloaca are material entities while the renal hilum and the ureterovesicular junction are immaterial ones. The relationships between embryological entities are described by standard ontology properties like *subClassOf* (left kidney is a subclass of kidney) and *part_of* (left kidney is a part of embryo) and its inverse *has_part*. We use also the property *develops_from* presented in [20] to describe the lineage between entities during the embryo development. Some entities *directly_develops_from* another, means that an organ is a direct outcome from another organ. This allows us to express for instance that the kidney *directly_develops_from* the metanephric blastema, the metanephric blastema *directly_develops_from* the metanephros and so, the kidney *develops_from* the metanephros (see inference section below).

### Temporal entity

Let us first note that we will use within this work the term evolution in its animation and modeling meaning, i.e. a surface undergoing geometrical deformation through time, and not with respect to its biological sense.

Temporal entities represent Carnegie stages and gestation weeks. These allow us to describe the evolution of the embryo at different granularities. In particular, the 23 Carnegie stages correspond to the first eight weeks of gestation. The remaining gestation weeks (form 9 to 12) are represented by single classes. Since Carnegie stages have different duration, we employ two new properties *from_gestation_day* to *to_gestation_day*, in order to describe the first and last day of each stage. For example, the stage 14 lasts two days (from day 32 to day 34), while the stage 17 lasts three days (from day 39 to day 42).

As we will discuss next, temporal entities are a crucial aspect of our ontology model for the procedural modeling of the embryo evolution. Moreover, each stage is described as a stage *following_stage* the previous one. For example, the stage te15 *following_stage* te14. This relation, *following_stage,* is simply used to express the chronological order between the gestation stages and it is similar to *immediately_preceded_by* found in RO (see Additional file 1: Table S1). A specific reasoning process over stages is presented in Osumi-Sutherland et al. [35] for automatic classification.

### Geometrical component

Geometrical components are classes representing 3D geometric shapes. These classes’ instances are paired with the spatio-temporal representation of organs. They allow the user to visualize the coarse representation of the organs at a user-prescribed time, i.e., Carnegie stage or gestation week. More specifically, we focus on being able to represent the overall shape, position, orientation and size, of the organs. We have defined the following 6 basic geometrical primitives to represent an organ or a part of it: point, line, plane, ovoid, cylinder and duct. We used only simple geometrical primitives in this work to enable the procedural generation of the geometry of organs and we explain in the discussion how to extend such representations to produce a more accurate visual representation of the organs.

**Fig. 1:**
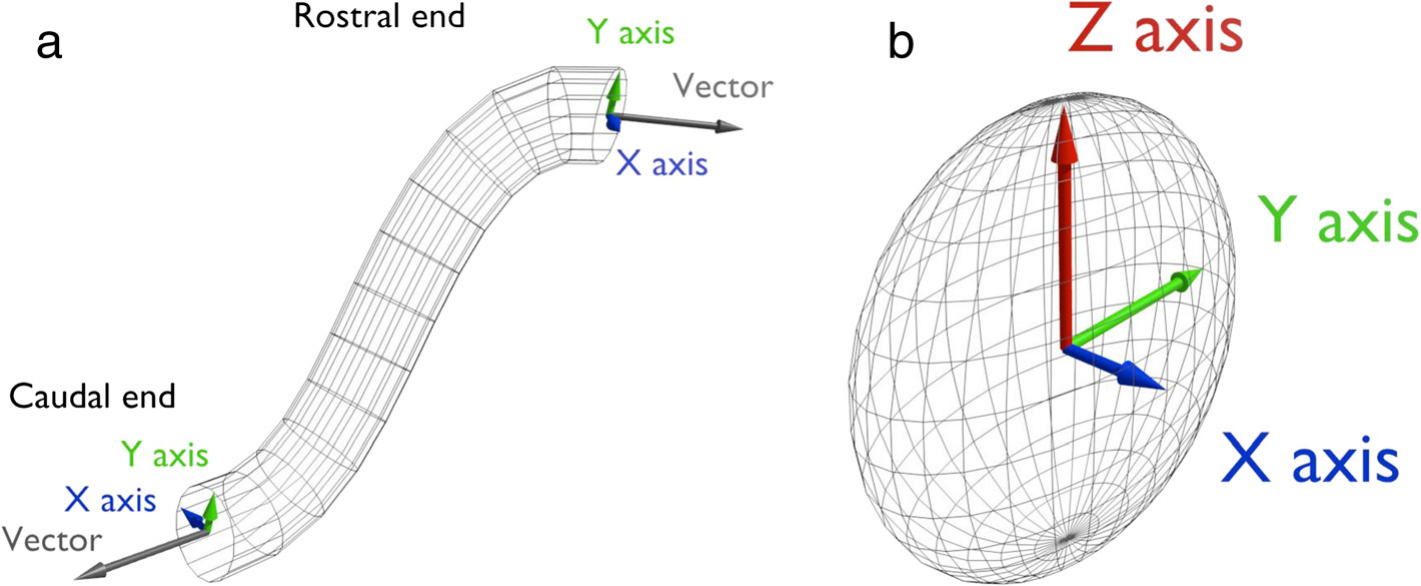
**a** Geometrical representation of a duct. The both rostral and caudal ends can be described and can have evolution separately. **b** Geometrical representation of an ovoid. This ovoid can be described using a barycenter and three axes with specific size and orientation

Ducts represent the ureter and other tubular structures. An example of such structure is given in Fig. 1a.

The planes are used to illustrate some immaterial embryological entities such as a foramen or embryo sagittal plane, or a flat side of an organ, e.g. the inferior side of liver.

Cylinders are used to describe some solid organs such as the diaphragm whose geometry is roughly cylindrical.

The line is used to describe a boundary of an organ or a particular geometric feature, e.g. the vertebral line, used to fix or to move an organ relatively to it.

Points are used to represent immaterial entities linking different organs, e.g. the left uretero-vesicular junction linking the ureter with the bladder**.** The contact area between two organs is described with a unique point belonging to both organs.

Of course, this set of geometric entities can be extended towards more complex geometric shapes.

Each geometric object is defined by a barycenter position, axis size, and vector orientation. For example, kidneys are drawn as ovoids (e.g. Fig. 1b). AT stages 14 and 15, we have two different instances of the ovoid class. Each instance provides information about the size of the ovoid axes, the position of the barycenter w.r.t. the embryo-axes and vector’s coordinates to orient the shape w.r.t. to the reference frame of the scene.

At the ontology level, these three informations are set with three properties, namely *axis_size*, *barycenter_position*, and *x, y and z_axis_orientation* that are subproperties of *vector_coordinates*. These properties are specialized further in order to process complex objects like ducts or lines. These two shapes have two endpoints, called the rostral and the caudal ends that must be described separately. Therefore we introduced some subproperties, namely *caudal_end_axis_size, rostral_end_axis_size, caudal_end_barycenter_position, rostral_end_barycenter_position, caudal_end_vector_coordinates, rostral_end_vector_coordinates.*

Finally, the property *has_geometrical_representation* is used to link the objects with the spatio-temporal representation of each structure, which are described next.

### Spatio-temporal representation

Spatio-temporal representations are the central part of our ontology. They establish a precise connection between the world of anatomy and 3D graphics, along the space and time dimensions.

The instances of this class link organs (e.g. the class *left_kidney*) with their geometrical representation (e.g. an instance of ovoid) and temporal entities (e.g. Carnegie stage 14). The property *describes* connects a spatio-temporal representation with an anatomical entity. Regarding the Xtemporal aspects, the property *at_stage* connects the spatio-temporal representation instance with a gestation moment. To describe the evolution of organs, spatio-temporal representation instances must also relate to time-intervals.

For example, to describe the process undergone by the ureter between stage 14 and 18, the process instance is explained by the property *has_process* that ties a spatio-temporal representation instance with a process instance as follows.

### Process

Evolutionary processes represent the observed phenomenon that can take place in the embryo during a gestation duration. We represented 6 kinds of processes: growth, migration, rotation, interaction, division and fixation.

Growth involves almost all organs. Migration involves organs like the kidney that moves from the pelvic to the lumbar region, or the gonad that does almost the same in the opposite direction. Rotation involves organs like the kidney that undergo a 90° rotation around two of its reference axes during its migration towards the lumbar region. Interaction between organs is necessary for their development like the ureter-kidney interaction, which is necessary for the kidney growth. Division can be either a physiological or a pathological process that leads an organ to abnormally split in different parts. At an example, it can be used to model the kidney duplication, occuring when a ureter (ureteric bud) splits too early before joining a kidney (metanephric blastema). Fixation describes the hard link of two anatomical entities.

We introduce some properties to describe the processes, for instance, the direction of a migration or a rotation of organs, the growth proportion, fixed-points for fixations, the interacting objects involved in a process. These properties are *migration_direction* (which is a subproperty of *vector_coordinates), rotation_degree, growth_proportion (*for *rostral* and *caudal ends), fixed_to (*for *rostral* and *caudal ends).*

Rostral and caudal subproperties allow a variation of the endpoints of an object like ducts and lines in a different way.

To capture the dependency between the different processes in the embryo evolution we also use the property *depends_on*. For example, the kidney growth depends on the interaction between ureter and kidney.

A spatio-temporal representation is linked to a process by *has_process* and to duration between two temporal entities using *from_stage* and *to_stage.*

It is important to note that the properties *starts_at, ends_at,* which are used to describe the existence of an organ over a time interval differ from the properties *from_stage* and *to_stage* which describe a process over a time interval. For example, the existence of the cloaca *starts_at* the Carnegie stage 11 and *ends_at* the Carnegie stage 14. However, the cloaca can have a process *from_stage* 11 *to_stage* 12 and another one *from_stage* 12 *to_stage* 14.

### Disease

The Disease class describes the embryo malformations through the activation or inactivation of some processes [36]. For instance, the hypoplastic kidney occurs when there is no interaction between the ureter and the kidney, and it inhibits the kidney growth. We use the property *impacts_processus* to relate diseases with impacted processes. We use the property *impacts_entity* to denote the anatomical entities involved in a process or impacted by a disease.

### Running example

We now describe in detail how the evolution of an organ is represented in our ontology. We will use the representation of the evolution of the left kidney during the period between Carnegie stages 14 to 20.

An instance of the organ left kidney is described through a set of spatio-temporal representations (3D). Below, e00_left_kidney is an instance of left kidney, i.e., the left kidney of a particular embryo (the embryo 00). The left kidney class is a *subClassOf* kidney, which is a *part_of* embryo.

**Fig. 2:**
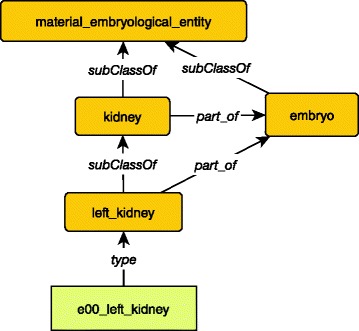
Representation of an organ in the ontology. Class and subclass are represented in dark orange round-angle boxes. Instances are represented in light orange right-angle boxes

The graphical representation of these triples is given in Fig. 2.

Concerning the 3D representation, the e00_left_kidney has a set of spatio-temporal representation instances, either at a given stage  or for a given duration .

**Fig. 3:**
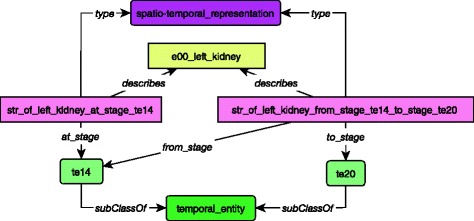
Links between spatio-temporal representation with organ instance and temporal entities. Each spatio-temporal representation is linked to a specific stage (*at_stage*) or to duration (*from_stage*, *to_stage*). The organs classes and instances are represented with orange boxes. Spatio-temporal representations are represented in purple boxes and temporal entities are represented with green boxes

This is depicted in Fig. 3.

**Fig. 4:**
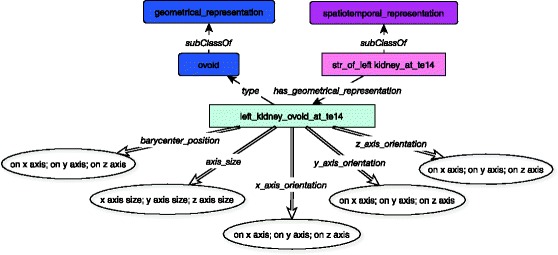
Static description of the left kidney at stage 14. Link between spatio-temporal representation and geometrical component (*has_geometrical_representation*). Each spatio-temporal representation is linked to a geometrical representation which allows to give some information about size, position and orientation of the organ. The double arrows show the link to dataproperties allowed to give some numerical information. The geometrical components are represented with blue boxes

The description of the kidney links its spatio-temporal representation instance to a geometrical component (*has_geometrical_representation*) during a specific stage (*at_stage).* This geometrical component is an ovoid which has some information about its *barycenter_position,* its *axis_size,* its *x, y and z_axis_orientation*, as illustrated in Fig. 4.

**Fig. 5:**
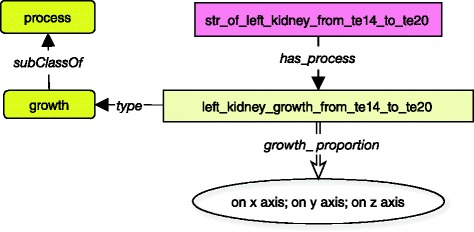
Dynamic description of the left kidney from stage 14 to stage 20. Each spatio-temporal representation for duration is linked to a process, which have some evolutionary characteristics, represented by double arrows to numerical information. Processes are represented in yellow boxes

The dynamic description of the left kidney is achieved through a connection between a spatio-temporal representation instance, a process (with property *has_process*) and a duration period (with properties *from_stage* and *to_stage*). For example, the fact that the left kidney has a *growth_proportion* of 10 on each of its three axes during the stages 14 to 20 of gestation is declared as in Fig. 5.

**Fig. 6:**
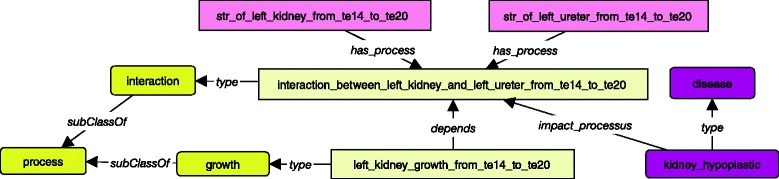
The impaction of a disease on a processus. A pathology can impact a process to lead to a malformation like a malrotation of an organ, a less of migration or growth. The pathologies are represented in dark purple boxes

We now illustrate how we represent pathologies. We consider the example of kidney hypoplastic pathology, which impacts the interaction between ureter and kidney during the embryo development, and thus the kidney growth process*.* We use the property *impacts_processus* to relate a pathology to the process it impacts (see Fig. 6).

When querying the ontology, we can discover that the kidney hypoplastic lacks a kidney growth.

**Fig. 7:**
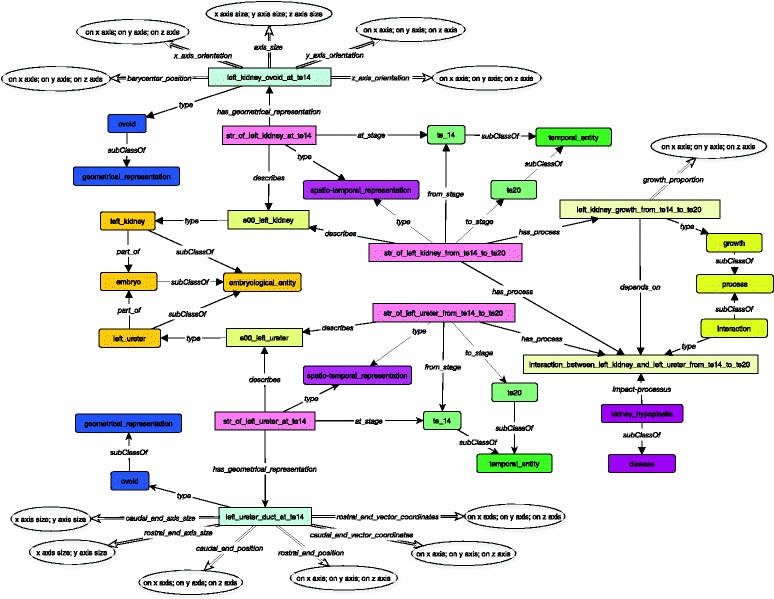
Overview of the left kidney static and dynamic descriptions in MyCF Embryo. For the kidney example, we can see the links with spatio-temporal representation, the geometrical component and the process, and the impact of the disease. Note the importance of the spatio-temporal representation which is the central part of our ontology

Below we illustrate the whole resulting ontology fragment (see Fig. 7).

### Inference

The inference rules of MyCF Embryo express complex connections between entities. These rules offer a uniform setting for expressing the semantics of most of the OWL and all the RDFS constraints (such as transitivity or symmetry of some generic properties like *subClassOf* or *subPropertyOf*, and of more specific properties like *part_of* or *develops_from*) but also domain-specific rules that have to be declared by the ontology designer. These rules capture in a very compact way implicit facts that can be made explicit on demand or at query time by an inference mechanism.

This mechanism is automatic and consists in applying the rules on the explicit facts declared and stored as RDF triples, in all the possible manners satisfying the conditions of these rules. For each possible instantiation of the variables (denoted by a name starting by ?) appearing in a condition part of a given rule such that all its conditions are satisfied by explicit facts, the new facts corresponding to the (appropriately instantiated) conclusion of the rule are added. This saturation process is iterated as long as new facts can be produced. The termination is guaranteed by the form of the rules that are considered. They correspond to *safe* rules, also called Datalog rules, i.e., all the variables appearing in the conclusion of a rule also appears in the condition part.

The rules that are considered in the current version of MyCF Embryo are summarized in Additional file 2: Table S2.

The first group of rules enriches the description of the embryological entities development. In particular, it describes the dependency between anatomical entities during the gestation by means of properties *directly_develops_from* and *develops_from* that denote direct and (possibly) indirect dependencies, respectively.

**Fig. 8:**
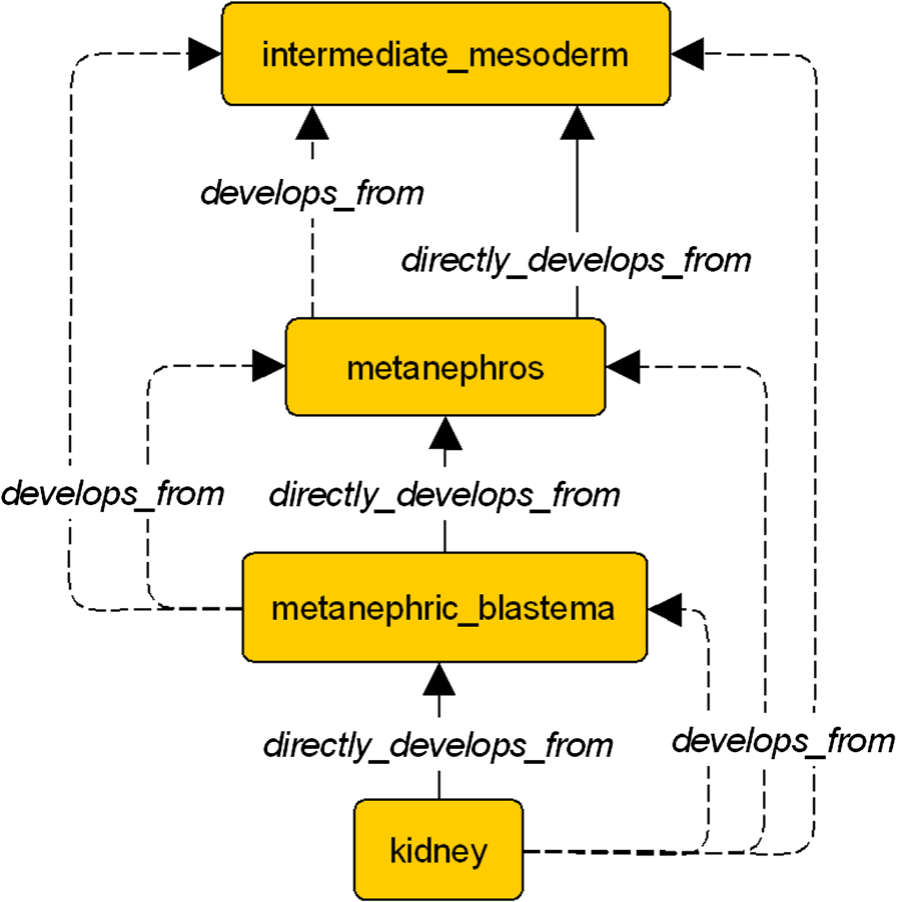
Inference R1 and R2. The inferred links are represented with dotted arrows

**Fig. 9:**
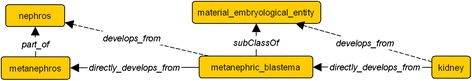
Inference R3 and R4

The rule R1 defines *directly_develops_from* as a subproperty of *develops_from*. This last one is a transitive property, as defined by rule R2. As illustrated in Fig. 8, rule R1 allows us to infer for instance that since the *kidney* directly develops from the *metanephric blastema*, then more generally it develops from that entity. Furthermore, because the *metanephric blastema* develops from the *metanephros*, by rule R2, we can infer that the *kidney* develops from the *metanephros*. Rule R3 describes the development relations between subparts of the embryo. As illustrated in Fig. 9, it allows for instance to infer that since the *metanephric blastema* directly develops from the *metanephros*, and this last one is a part of the *nephros*, then the *metanephric blastema* develops from the *nephros*. Rule R4, is the analogous of rule R3, for the subClassOf property. It is also illustrated in Fig. 9.

The second group of rules describes the relations between embryological entities and geometrical entities through their spatio-temporal representations at different gestation stages.

The rule R8 allows to tie embryological entities with their geometric representations, so as to answer to queries like "*which geometrical representations of kidneys are available?*”.

**Fig. 10:**
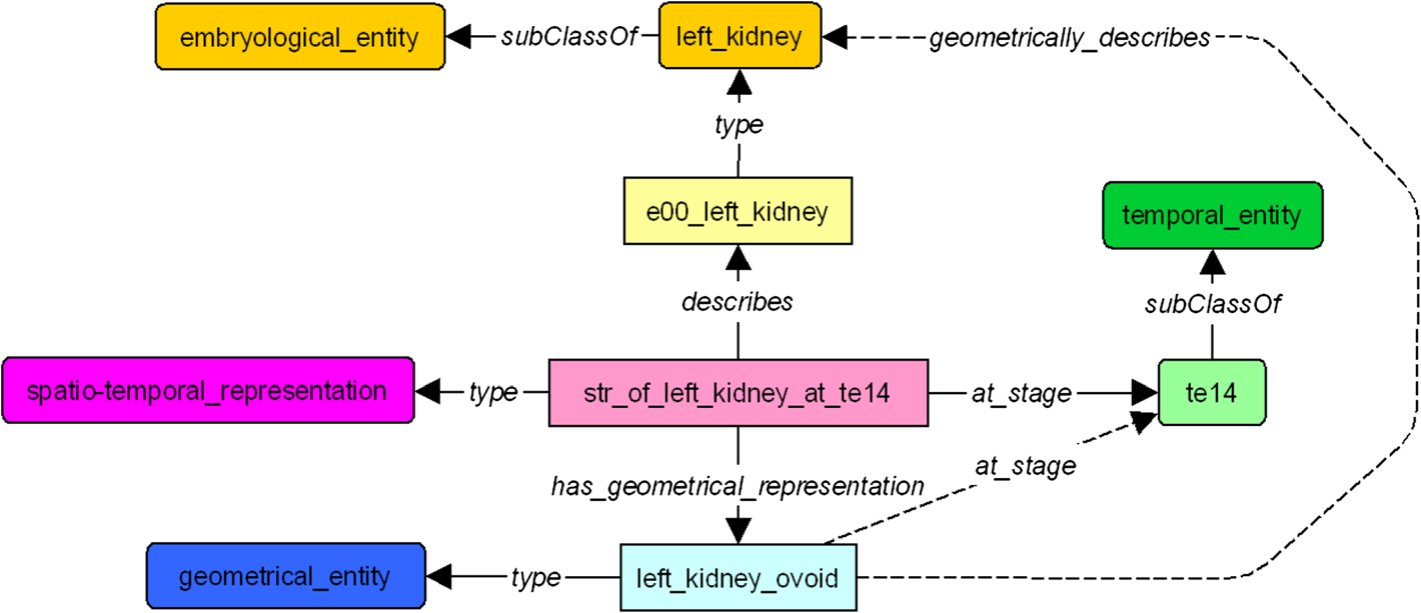
Inference R8 and R9. Str correspond to the spatio-temporal representation instance

The effect of this rule is illustrated in Fig. 10. It allows us to infer that a given instance *e00_left_kidney* of the *left_kidney* entity can be described by a specific *left_kidney_ovoid* geometrical model, associated to its spatio-temporal representation *st_representation_of_left_kidney_at_te14*.

The rule R9 further describes geometrical representation of an embryo organ by explicitly inferring the gestation stage of the organ that it represents. As shown in Fig. 10 it allows us to infer that since *st_representation_of_left_kidney_at_te14* corresponds to the Carnegie stage 14 (te14), then the corresponding geometrical model *left_kidney_ovoid* describes the corresponding instance of left kidney at this stage 14.

At this point, it is also possible to query the ontology and ask for "*all geometrical representations (?g) of entities (?ee) that develop from the Cloaca, starting from stage 14*". This translates to the following SPARQL query

To impose a discrete order between the gestation stages, we introduced the property *following_stage*. This permits to state that stage 15 follows stage 14 and that stage 16 follows stage 15. By means of the rule R10, we can compute the total order between the gestation stages, so as to infer also that stage 16 follows stage 14, and have a complete answer to our query.

**Fig. 11:**
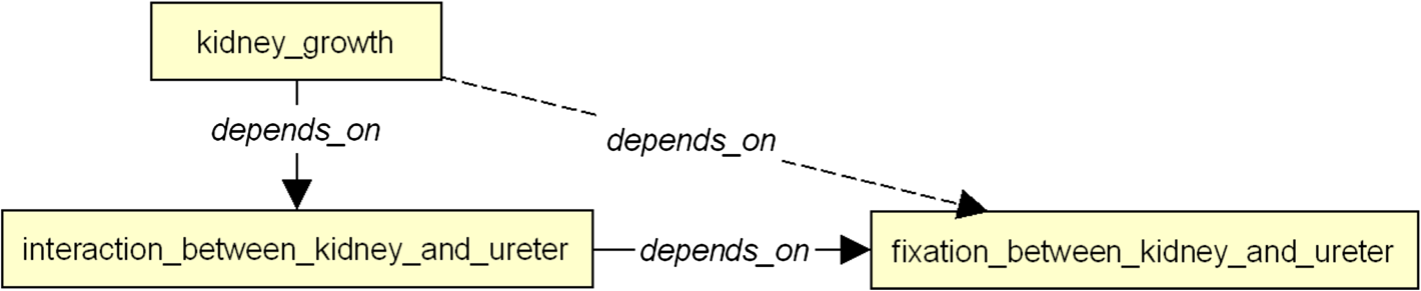
Inference R11

**Fig. 12:**
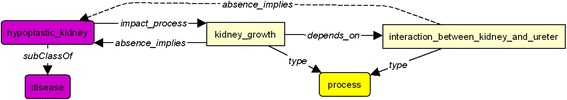
Inference R12

**Fig. 13:**
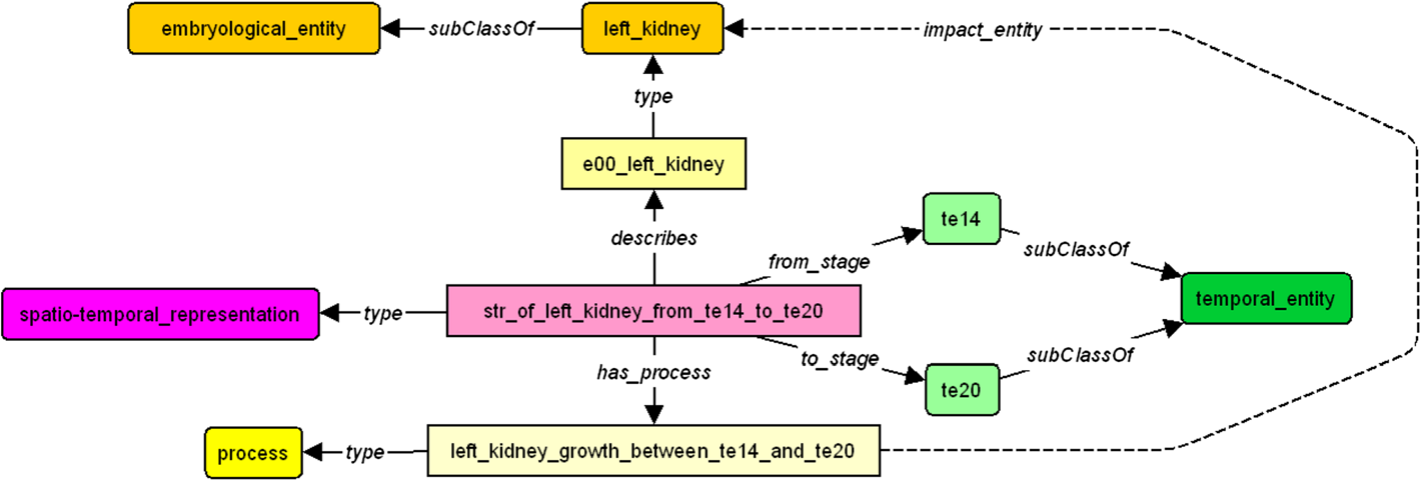
Inference R13

**Fig. 14:**
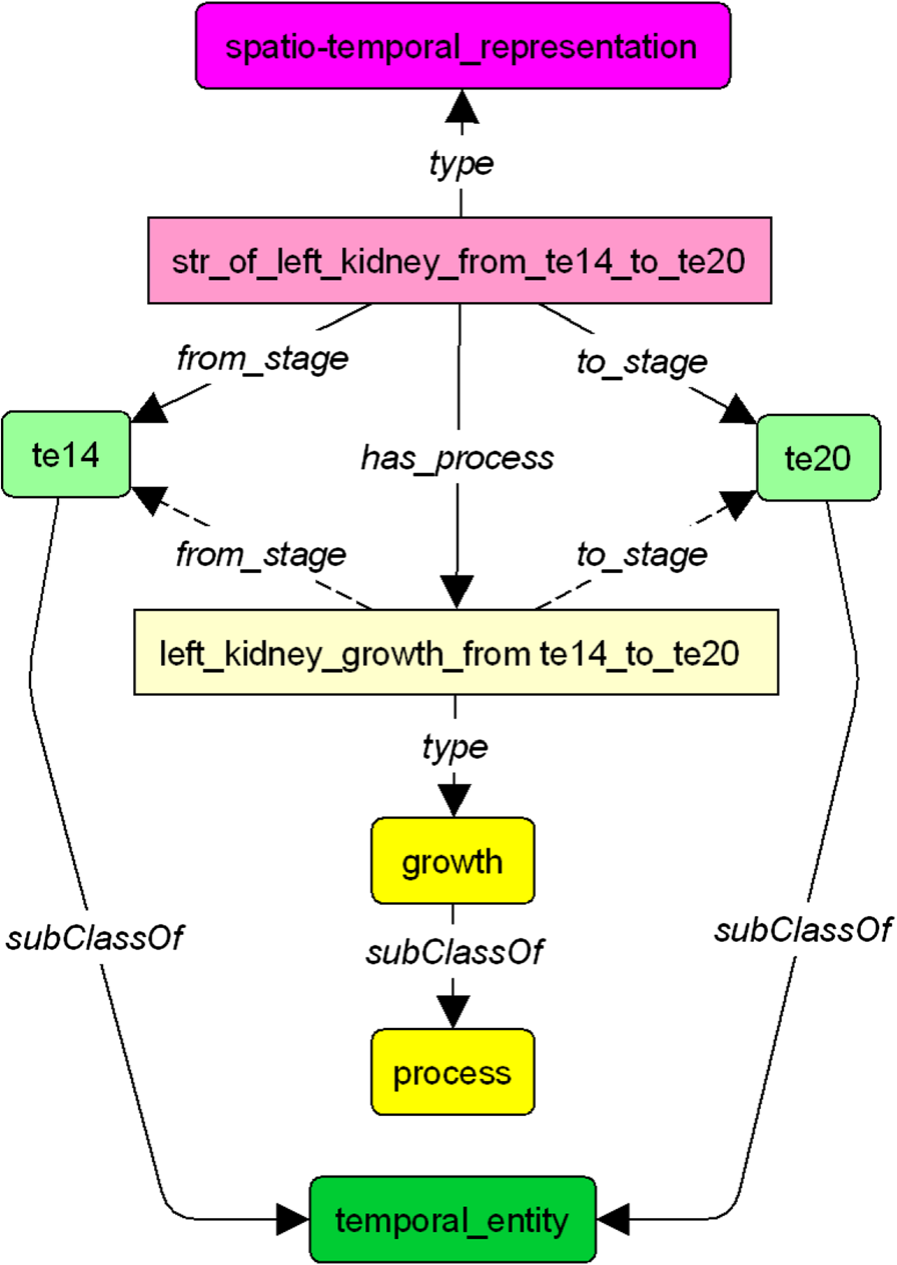
Inference R14 and 15

The last group of rules enriches the description of the evolution processes of the embryo. Rule R11 defines the indirect dependency between processes. As illustrated in Fig. 11, as the *kidney growth* depends on the *interaction between kidney and ureter* and this last one depends on the *fixation between kidney and ureter*, we can deduce that *the kidney growth* depends on the *fixation between kidney and ureter.* Rule R12 connects processes and pathologies. For example, as shown in Fig. 12, since the lack of *kidney growth* implies the *hypoplastic kidney,* and that the *kidney growth* depends on the *interaction between kidney and ureter,* we can also deduce that the lack of *interaction between kidney and ureter* implies the *hypoplastic kidney*. The last two rules relate processes to the anatomical entities they impact. As depicted in Fig. 13, rule R13 allows us to infer that process *left_kidney_growth_between_te14_and_te20* impacts entity *left_kidney* due to the fact that the *left_kidney_growth_between_te14_and_te20* is the process described by the spatio-temporal representation *st_representation_of_left_kidney_from_te14_to_te20*, and this spatio-temporal representation describes an instance of the *left_kidney*. Finally rule 14 and 15 are used to infer the Carnegie stages where a process occurs. These are illustrated in Fig. 14. For example, we can infer that the process *left_kidney_growth* occurs between stages 14 and 20, because its associated spatio-temporal representation lasts during this period.

### 3D visualization and modeling

Finally, the animated 3D model is built using the information stored into the ontology. The scene creation requires three steps. First, the user selects the organs and the gestation period. Secondly, all the informations describing those organs are retrieved from the ontology. Thirdly, these informations are used to create the meshes corresponding to the organs, and to animate the scene.

For example, the user can select a set of organs of the uro-genital system (gonads, kidneys, ureters, cloaca, bladder, rectum), and a period which goes from Carnegie stage 10 to gestation week 12.

The following query extracts all geometrical representations and properties of the left kidney for the reference model we built, named mcfe: e00_left_kidney.

For each spatio-temporal representation (?str) and geometrical representation (?ge) of the left kidney, this query extracts the geometrical shape (?shape), the stage (?stage) and all informations needed to describe the shape of organs (?property and ?value) like their axis size or their barycenter location.

Informations about processes are obtained in a similar way.

The organ information extracted from the ontology is procedurally interpreted. The first step is to create a static scene containing all the selected organs. Each organ of the scene is a mesh generated with the information obtained from its geometrical entity in the ontology.

We now describe how the meshes are generated. When the geometrical shape is an ovoid or a cylinder, the mesh can be simply generated from the ovoid or cylinder barycenter position, axis size and orientation. When the geometrical shape is a duct, the mesh is generated as a sequence of cylinders around its skeletal line. This skeleton is defined by a Bézier curve, whose endpoints coordinates and tangents are directly given in the ontology. In some cases, a complex geometry may not be fully procedurally described. In such a case, an existing mesh can be specified as a link in the ontology. Therefore, the mesh can be included directly in the 3D scene.

**Fig. 15:**
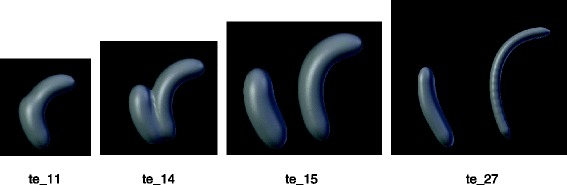
3D geometrical modelization of the cloaca’s division process is driven by the ontology

**Fig. 16:**
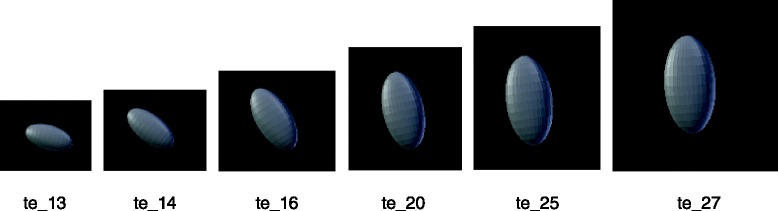
Example of the growth and rotation process on two axes of the kidney

**Fig. 17:**
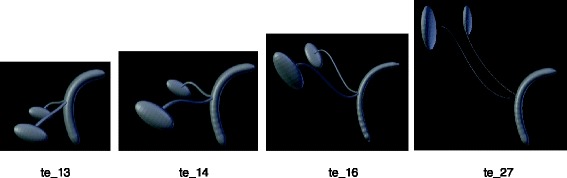
Example of the migration, rotation, growth of kidneys and ureters

Fusion and division processes require specific modeling approaches because the associated shape of organs must blend or split seamlessly. For example, the cloaca divides itself into two parts becoming the bladder and the rectum. These organs are represented using implicit surface modeling which are surfaces defined as the constant value, called isovalue, of a field function defined in 3D space. In our implementation, we define the field function as a smooth decreasing function of the distance to a reference point, or a reference curve, called the skeleton of the implicit shape. More specifically, we chose to use a polynomial field function with compact support called meta-ball. Finally, the implicit surfaces are converted into a mesh for the visualization (Figs. 15, 16 and 17).

Lastly, the scene is procedurally animated, thanks to the information given by the evolution processes. As the Carnegie stages may have different durations, we consider that the gestation day is the common unit of time to compute the animation through all stages. As an example, stage 11 lasts one day whereas stage 17 lasts three. Consequently, the animation of stage 11 lasts one time unit whereas stage 17 lasts three.

Given these settings, it is possible to generate an animated 3D scene representing the embryo development given only high-level descriptions of the contributing organs. To illustrate how a 3D scene and an animation of a complex process (such a kidney and urogenital formation and migration with ureter fixation) can be built and monitored by the ontology. The complementary video (see Additional file 3: movie 1) firstly shows each step described in the ontology in an independent manner, and lastly the resulting complex 3D animation. This video demonstrates the effective monitoring of the ontology over the 3 D models of organs displayed using a basic rendering. High quality rendering of the scene could be explored in a future work.

## Discussion

This work proves that a 3D graphical model can be monitored by an appropriate ontology. This ontology incorporates a subset devoted to spatial knowledge, which allows the description of organs as 3D shapes, and another subset focusing on temporal knowledge describing these organs at a specific stage. This ontology is also deductive, i.e., from an initial condition and some evolutionary process, it can compute a final condition through a procedural approach. As an example, an initial position and orientation of the kidney at stage 14 with two evolutionary processes (growth and rotation) effectively produces its rotational movement and corresponding growth.

This work aims at making a step towards automatic and adaptive 3D modeling and simulation of complex embryological processes. This distinguishes our work from existing 3D atlases in which ontologies are mainly used to navigate through static, predefined 3D illustrations.

Our 3D geometrical representation of the organs is limited to simple 3D primitives. We explain hereafter how this work could be extended to handle more accurate 3D shapes.

Firstly, let us note that the organs can be described hierarchically (see for instance Palombi et al. [21,22]). Secondly, complex and detailed shapes can be defined and deformed with the knowledge of a more simple one acting as proxy or bounding structure. This implies that a 3D scene describing the evolution of the organs can be described using two levels of details.

At the scale of the overall scene, the purpose is the definition of the global interactions between different organs. For instance, these interactions can be the relative position and collisions between organs, or the branching structures. At this level, all the organs can take part to the computation, and the 3D local geometry may not need to be accurate. Note that this level is the one we described in this paper using simple primitives.

At the complementary level, the organs are described more accurately, possibly using lower levels of details. These levels that we plan to address in a future work can be handled more locally and may involve only a single organ at a time, or the direct neighboring organs in the case of collisions. We propose to handle this local geometrical description in interleaving two approaches. Firstly, using a parametric description incorporating the use of more basic shapes associated with more parameters handled within the ontology. Secondly, using a mesh based representation to achieve accurate 3D description and visualization. Such a mesh will use the knowledge of the coarse representation acting as a guiding structure to be deformed. The deformation itself can be implemented using a smooth volumetric interpolation function such as mean value [37], harmonic [38] or Green [39] coordinates functions, for instance. Note that simulation-based approaches and self-collision handling can also be used with the previous approaches to handle small geometrical details.

We believe that using such an approach, i.e. interleaving the basic guiding shapes with the complex mesh description in an iterative process applied through the different level of details can converge toward a more accurate geometrical description of the organs.

We can also note that, introducing a physically-based simulator in the 3D model could extend the range of simulated events for pathological studies. More specifically, the research and teaching of teratology "in silico" could benefits from the proposed approach. Indeed, the complexity of these topics makes the proposed 3D representation essential for a good understanding.

## Conclusion

We have demonstrated that an ontology can be used to unify and organize medical knowledge in the domain of embryo development together with 3D modeling processes and animation.

Also we have shown the extensibility and scalability of our declarative approach based on RDF triples and rules. In fact, this work extends the ontology MyCF [21] which has been developed and enriched by medical students who had no difficulty after a short training to edit and update a RDF-based knowledge base. The scalability of the approach had already been shown for MyCF that contained about 74000 classes and properties as much as 11 rules for describing 3D models of human body and its functions.

We intend to extend the ontology for all structures in the embryo for the entire duration of gestation and to validate the ontology using information from scans of real embryos. All ontologies represent the knowledge of a community at a given time, and must be continually updated and improved to remain up to date with the latest knowledge. A unique feature of our approach is the declarative nature of the graphical models, which makes it possible for domain experts to enrich the knowledge base at any time, through simple edit operations without having to modify the (domain-independent) reasoning algorithmic machinery used for answering queries. Embryology is in constant evolution, benefiting from other sciences progress. We aim at making a step toward a collaborative tool that will ultimately improve medical care.

## Material and methods

The main purpose of this study was to generate an ontology of human developmental anatomy unifying embryology and 3D modeling processes.

Our ontology describes in a comprehensible way the different geometrical informations, evolution and interactions between various organs during gestation. These descriptions must be formatted in a way such that a 3D model can be procedurally generated using information solely queried from the ontology. Our objective was not to build another ontology as other already exist, rather it was to add geometrical and dynamic information to existing ontologies enabling powerful applications in 3D graphics and visualization domains.

In this work, two tasks have been addressed simultaneously. Firstly, we have built an ontology including all the required information. To achieve this, we created different categories that describe entities' geometrical states and evolution, and a set of properties to link the different parts together. Secondly, we developed a software tool that generates a 3D graphical scene representing the embryo development. To do so, it queries the ontology and extracts all information necessary for modeling, visualizing and simulating the 3D scene.

We focused on the development of the urinary system. We believe this system to be a good study case as its development involves a diversity of mechanisms that are representative of other evolution processes and a large variety of diseases caused by anomalies of these processes.

We used the TopBraidComposer [40] tool which allows the creation and edition of lightweight ontologies in RDF format [41] format lightweight ontologies, and to query them using SPARQL [42].

RDF and SPARQL are standards recommended by the W3C for the semantic Web and Linked Data \footnote{http://linkeddata.org/}.

In addition, TopBraidComposer allow the addition of inference rules on top of RDF datasets and supports the application of these rules on RDF facts until saturation (i.e., until all the possible facts that can be inferred using the rules have been obtained). RDF datasets equipped with rules allow the capture of most of OWL \cite{owl} constraints that are useful in practice, such as the transitivity or symmetry properties, as well as domain-specific rules with practical relevance for users in many domains of interest. The 3D graphical tool was developed using the 3D open source software Blender [43]. All the 3D surfaces where procedurally handled using Python [44] scripting within the Blender framework. The ovoids were handled as Blender parameterized primitives. The implicit surfaces were represented as a set of points along the trajectory of the ducts while the field function and the representative mesh of the isovalue was handled by Blender software. Finally, the shape representing the boundary of the embryo was created interactively using Blender interface. All the coordinates parameterizing the shapes and its animation were expressed locally with respect to the embryo main axes and size. The coordinates were queried within our Python script to be directly usable within the Blender framework.

Our ontology is public available: [https://mybody.inrialpes.fr/mycfembryo/].

## Additional files

Additional file 1: Table S1. It contains the detail of each used properties, the number of properties, classes and instances of the ontology. (DOCX 24 kb) Additional file 2: Table S2. It contains the rules that are considered in the current version of MyCF Embryo. (DOCX 14 kb) Additional file 3: Movie 1. It demonstrates the control of 3D modeling by the ontology. (MOV)
