# Supplementary material for: My Corporis Fabrica Embryo: An ontology-based 3D spatio-temporal modeling of human embryo development
Source: J Biomed Semantics. 2015 Sep 24;6:36. doi: 10.1186/s13326-015-0034-0 (PMC4582726; doi:10.1186/s13326-015-0034-0)
Supplement: Additional file 1: Table S1. — It contains the detail of each used properties, the number of properties, classes and instances of the ontology. (DOCX 24 kb) [file 13326_2015_34_MOESM1_ESM.docx]

**Table 2.**

**The rules of MyCF Embryo**

| **Rules** | **Meaning** |
| --- | --- |
| **R1: if** (?x directly_develops_from ?y) **then** (?x develops_from ?y)  **R2: if** (?x develops_from ?y), (?y develops_from ?z) **then**  (?x develops_from ?z)  **R3: if** (?x directly_develops_from ?y), (?y part_of ?z) **then**  (?x develops_from ?z)  **R4: if** (?x develops_from ?y), (?y subClassOf ?z) **then**  (?x develops_from ?z)  **R5: if** (?x part_of ?y), (?y part_of ?z) **then** (?x part_of ?z)  **R6: if** (?x subClassOf ?y), (?y subClassOf ?z) **then** (?x subClassOf ?z)  **R7: if** (?x type ?y), (?y subClassOf ?z) **then** (?x type ?z) | “directly_develops_from” is a subproperty of “develops_from” *(expressible in OWL and RDFS)*  “develops_from” is transitive *(expressible in OWL)*  “directly_develop_from” composed with “part_of” is a way to infer new “develops_from” facts  ( *expressible in OWL as a property chain axiom*)  “directly_develop_from” composed with “subClassOf ” is another way to infer new “develops_from” facts (*domain-specific rule not expressible in OWL*)  “part_of” is transitive *(expressible in OWL)*  “subClassOf” is transitive *(standard OWL and RDFS semantics)*  inheritance of instances between classes *(standard OWL and RDFS semantics)* |
| **R8:**  **if** (?e type ?ee), (?ee subClassOf embryological_entity),  (?str describes ?e) , (?str has_geometrical_ representation ?g)  **then** (?g geometrically_describes ?e)  **R9: if** (?str has_geometrical_representation ?ge), (?str at_stage ?te)  **then** (?ge at_stage ?te)  **R10**: **if** (?x following_stage ?y), (?y following_stage ?z)  **then** (?x following_stage ?z) | defines a shortcut relation “geometrically_describes” between a geometrical representation (?g) and an embryological entity (?e) as soon as this geometrical representation ?g is associated with a given spatio-temporal entity (?str) that “describes” ?e  (*domain-specific rule not expressible in OWL*)  this rule associates the gestation stage corresponding to a given spatio-temporal entity (?str) to its geometrical representation (?ge)  (*domain-specific rule not expressible in OWL*)  “following_stage” is transitive *(expressible in OWL)* |
| **R11:**  **if** (?x depends_on ?y), (?y depends_on ?z) **then** (?x depends_on ?z)  **R12: if** (?x absence_implies ?y), (?x depends_on ?z)  **then** (?z absence_implies ?x)  **R13: if** (?str describes ?x), (?str has_process ?p), (?x type ?ee)  **then** (?p impact_entity ?ee)  **R14: if** (?str has_process ?p), (?str from_stage ?te) **then** (?p from_stage ?te)  **R15: if** (?str has_process ?p) and (?str to_stage ?te) then (?p to_stage ?te) | “depend_on” is transitive *(expressible in OWL)*  transitivity of relation “absence_implies” through the relation “depend_on” (*not expressible in OWL*)  defines the relation “impact_entity” in function of the relations “describes” and “has_process”  (*domain-specific rule not expressible in OWL*)  transitivity of relation “from_stage” through the relation “has_process” (*not expressible in OWL*)  transitivity of relation “to_stage” through the relation “has_process” (*not expressible in OWL*) |
