# Supplementary material for: My Corporis Fabrica Embryo: An ontology-based 3D spatio-temporal modeling of human embryo development
Source: J Biomed Semantics. 2015 Sep 24;6:36. doi: 10.1186/s13326-015-0034-0 (PMC4582726; doi:10.1186/s13326-015-0034-0)
Supplement: Additional file 2: Table S2. — It contains the rules that are considered in the current version of MyCF Embryo. (DOCX 14 kb) [file 13326_2015_34_MOESM2_ESM.docx]

https://drive.google.com/folderview?id=0B8nk7axMn1hDfmlrbE9OV05qODJua1hfeDNab0tNVVpJTE1scjVxZG1WNFlVWDhlRnZzcVE&usp=sharing
